# Neuraminidase-associated plasminogen recruitment enables systemic spread of natural avian Influenza viruses H3N1

**DOI:** 10.1371/journal.ppat.1009490

**Published:** 2021-04-23

**Authors:** Jacob Schön, Angele Breithaupt, Dirk Höper, Jacqueline King, Anne Pohlmann, Rokshana Parvin, Klaus-Peter Behr, Bernd-Andreas Schwarz, Martin Beer, Jürgen Stech, Timm Harder, Christian Grund

**Affiliations:** 1 Institute of Diagnostic Virology, Greifswald–Insel Riems, Germany; 2 Department of Experimental Animal Facilities and Biorisk Management, Greifswald–Insel Riems, Germany; 3 Department of Pathology, Bangladesh Agricultural University, Mymensingh, Bangladesh; 4 AniCon Labor GmbH, Höltinghausen, Germany; 5 Vaxxinova Diagnostics GmbH, Leipzig, Germany; 6 Institute of Molecular Virology and Cell Biology, Greifswald–Insel Riems, Germany; Johns Hopkins Bloomberg School of Public Health, UNITED STATES

## Abstract

Repeated outbreaks due to H3N1 low pathogenicity avian influenza viruses (LPAIV) in Belgium were associated with unusually high mortality in chicken in 2019. Those events caused considerable economic losses and prompted restriction measures normally implemented for eradicating high pathogenicity avian influenza viruses (HPAIV). Initial pathology investigations and infection studies suggested this virus to be able to replicate systemically, being very atypical for H3 LPAIV. Here, we investigate the pathogenesis of this H3N1 virus and propose a mechanism explaining its unusual systemic replication capability. By intravenous and intracerebral inoculation in chicken, we demonstrate systemic spread of this virus, extending to the central nervous system. Endoproteolytic viral hemagglutinin (HA) protein activation by either tissue-restricted serine peptidases or ubiquitous subtilisin-like proteases is the functional hallmark distinguishing (H5 or H7) LPAIV from HPAIV. However, luciferase reporter assays show that HA cleavage in case of the H3N1 strain in contrast to the HPAIV is not processed by intracellular proteases. Yet the H3N1 virus replicates efficiently in cell culture without trypsin, unlike LPAIVs. Moreover, this trypsin-independent virus replication is inhibited by 6-aminohexanoic acid, a plasmin inhibitor. Correspondingly, *in silico* analysis indicates that plasminogen is recruitable by the viral neuraminidase for proteolytic activation due to the loss of a strongly conserved N-glycosylation site at position 130. This mutation was shown responsible for plasminogen recruitment and neurovirulence of the mouse brain-passaged laboratory strain A/WSN/33 (H1N1). In conclusion, our findings provide good evidence in natural chicken strains for N1 neuraminidase-operated recruitment of plasminogen, enabling systemic replication leading to an unusual high pathogenicity phenotype. Such a gain of function in naturally occurring AIVs representing an established human influenza HA-subtype raises concerns over potential zoonotic threats.

## Author summary

Avian influenza viruses (AIV) of subtype H3N1 caused outbreaks among gallinaceous poultry associated with an unusual high mortality in Belgium in 2019. Although those strains shared properties of a high pathogenicity (HP)-like phenotype in chicken experiments, still the viral hemagglutinin endoproteolytic cleavage site (HACS) matches that of canonical low pathogenicity (LP) AIV. Above all, the HACS motif is the molecular marker to distinguish low from high pathogenicity phenotypes in H5 and H7 AIV. Monobasic HACS motifs restrict the LPAIV to use tissue-specific host proteases and therefore confine their organ tropism to the respiratory and digestive tracts. By contrast, the polybasic HACS of HPAIV provides access to the ubiquitously expressed prohormone convertase furin, resulting in a grossly broadened organ tropism. Here, we show that the systemic spread of the Belgian H3N1 chicken viruses is independent from tissue-specific proteases and that their proteolytic HA activation is processed by plasminogen and associated to the loss of a conserved N-glycosylation site at position 130 of the neuraminidase. To date, this mechanism was only described for the mouse-adapted laboratory H1N1 strain WSN/33. Our data, however, provide evidence that this mechanism can also evolve in natural circulating AIV, as well as in human strains, suggesting a certain zoonotic potential.

## Introduction

Influenza A viruses (IAV), orthomyxoviruses with an eightfold segmented genome, are widespread in the animal kingdom. Wild birds in the orders Anseriformes and Charadriiformes are considered a large natural reservoir of IAV comprising all of the sixteen hemagglutinin (HA; H1-16) and nine neuraminidase (NA; N1-9) subtypes in avian strains [1–6]. Whereas the majority of avian Influenza viruses (AIV) are of low pathogenicity (LPAIV), i.e., they induce no or only mild clinical signs after oculonasal or even intravenous (i.v.) inoculation of chickens, some strains of the subtypes H5 and H7 are of high pathogenicity (HP), causing rapid death in chicken with systemic spread [7]. As a major determinant for high pathogenicity, a polybasic motif within the endoproteolytic cleavage site of the HA was recognized [8], whereas the LPAIV carry a monobasic hemagglutinin cleavage site (HACS).

Our investigations were sparked by reports of a widespread LPAIV H3N1 epizootic in poultry comprising 82 chicken and turkey flocks in Belgium over four months [9,10] and three outbreaks in France [11]. Whereas the mortality was as high as 60%, suggesting the causative agents to be HPAIV, the identified AI viruses carried a typical monobasic HACS, indicating an LPAIV. Accordingly, their pathogenicity in chicken after intravenous inoculation was low with intravenous pathogenicity indices (IVPI) of 0.13 and 0.28, respectively, for two representative isolates [9,10]. However, consistent with field observations, clinical signs and course of disease could be reproduced in 34-week-old SPF layer hens (58% mortality and 100% egg drop, n = 24) [10] and in 24-week-old conventional Bovans Brown layer hens (33% mortality, n = 6) [9]. Besides, PCR analyses of organs from the latter birds supported the notion of systemic virus spread, including dissemination to the brain [9]. Those surprising results, along with such unusual field observations of lesions e.g. point bleedings in the brain [9], are very uncommon for LPAIV and rather indicative of HPAIV. Therefore, they prompted us to address the neurovirulence and underlying pathomechanisms which facilitate systemic replication of these viruses. Our initial IVPI experiments with two virus isolates from chicken (CK/BE/1940/19) and turkey (TK/BE/1358/19) revealed disorders of the central nervous system (CNS) in one out of ten birds per group. Therefore, in search of an explanation, we then thoroughly investigated the neurotropism by intracerebral (instead of intravenous) inoculation of chicks, and further pursued the question of an alternative mechanism of proteolytic HA activation.

To become functionally active, the HA precursor (HA_0_) has to be cleaved by host proteases into the disulfide-linked HA_1_ and HA_2_ subunits [12,13]. Only the cleaved form is able to undergo conformational changes at low pH in endo-lysosomal vesicles following attachment. This change is required to expose the highly conserved fusion peptide at the N-terminus of the HA_2_ subunit, which then initiates fusion of the viral with the endosome membranes, resulting in intracytoplasmic release of the viral genome (reviewed in [14]). The HA_0_ cleavage site for mammalian strains and LPAIV includes a single arginine or lysine at the C-terminus of the HA_1_, designated as position P1. This monobasic HA cleavage site is assessed by trypsin-like proteases, whereas their physiological tissue distribution is a major constraint of the viral organ tropism [12,13,15]. *In ovo*, the replication of LPAIV is restricted to the entodermal layer of the chorioallantoic membrane (CAM) of the chicken embryo [16]. In contrast, HPAIV viruses are able to invade the mesodermal and ectodermal layer of the CAM leading to subsequent systemic spread. This phenomenon is due to the presence of a polybasic cleavage site (pHACS; motif RXK/RR (P4-P1)) that is cleaved by the ubiquitous prohormone convertase furin and hence grossly broadens viral tissue tropism throughout the bird including endothelial, myocardial and neuronal cells [17]. LPAIV infection *in vivo* is restricted to the respiratory and digestive tract with only mild or no clinical disease. After oculonasal inoculation, virus replication is initiated in the epithelium of the choanal cavity, extending to the trachea and air sacs as well as the intestinal tract [18,19]. In addition, following i.v. inoculation, some strains replicate in renal tubules and pancreatic acinar cells [20,21]. In wild birds, the infection is subclinical and usually only detected in active surveillance studies [22,23]. In poultry (gallinaceous birds kept for egg or meat production), infection is associated with no or only mild clinical disease of the upper respiratory tract and/or drop in egg production [24–27]. Daily mortality rates above 0.08% for indoor layer chickens and 0.13% for outdoor layer chickens, together with weekly egg-production ratio of 0.94 could give indications for LPAIV infection [28]. However, in particular H9N2 or H6NX infections with secondary bacterial infections may lead to exacerbated disease and increased mortality rates of up to 65% [29–31]. Because secreted bacterial proteases can also activate the HA_0_ of LPAIV, those enzymes may aggravate the local infection but do not broaden the organ tropism of LPAI viruses [32]. However, there is evidence that viruses with monobasic HACS are able to affect additional organs beside the respiratory and intestinal tract. Some H10 AIV exhibit an HP phenotype, even though they still harbor a monobasic HACS [33,34]. Here, the pathology in chickens is dominated by renal failure which is observed, in particular, after i.v. inoculation. Moreover, H10 strains were described to spread to the brain [35]. Remarkably, neurotropism was also found in humans infected with seasonal IAV that carry a monobasic HACS [36,37] and for the A/WSN/33 H1N1 strain in mice [38]. The latter is derived from the first human IAV isolate [39] and was generated by numerous passages *inter alia* in chicken and mice brains [38]. It replicates without trypsin supplementation *in vitro* and exhibits neurovirulence and high pathogenicity in mice [40–42]. Further research identified NA-mediated plasminogen recruitment as causative for proteolytic cleavage of the HA_0_ precursor protein and further correlated this ability with trypsin-independent *in vitro* growth and neurovirulence [40,43–46]. Plasminogen is the zymogen of the serine protease plasmin, which is essential in fibrinolysis [47]. It is synthetized in the liver and circulates in the blood stream while it is present in most organs to different extents, too [48]. For the WSN NA, plasminogen binding was attributed to the substitution of an asparagine in a conserved N-linked glycosylation sequon at amino acid position 130 (WSN-NA N130R) and a C-terminal lysine [40,43,44]. However, the related H1N1 strain from 1918 was shown to recruit another unknown protease, enabling the virus to replicate in absence of trypsin in mammalian but not in avian cells [49]. For some other strains a NA-independent way of plasminogen recruitment is mediated by host annexin-2, incorporated in the virus membrane [50]. Therefore, IAV replication independent of trypsin *in vitro* is considered an important indicator for pathogenicity in mammals [51], whereas mechanisms enabling replication of LPAIV in the brain of birds have not been resolved so far.

Here, we found proteolytic HA activation by plasmin most likely NA-mediated, previously only identified in the laboratory generated strain WSN, in circulating chicken H3N1 LPAIV. Beyond that, our results establish the loss of the N-glycosylation site at NA 130 to be an important molecular marker for neurovirulence and systemic spread of natural avian influenza viruses.

## Results

### Belgian H3N1 occasionally expressed neurotropism in juvenile chickens following intravenous inoculation

**Fig 1:**
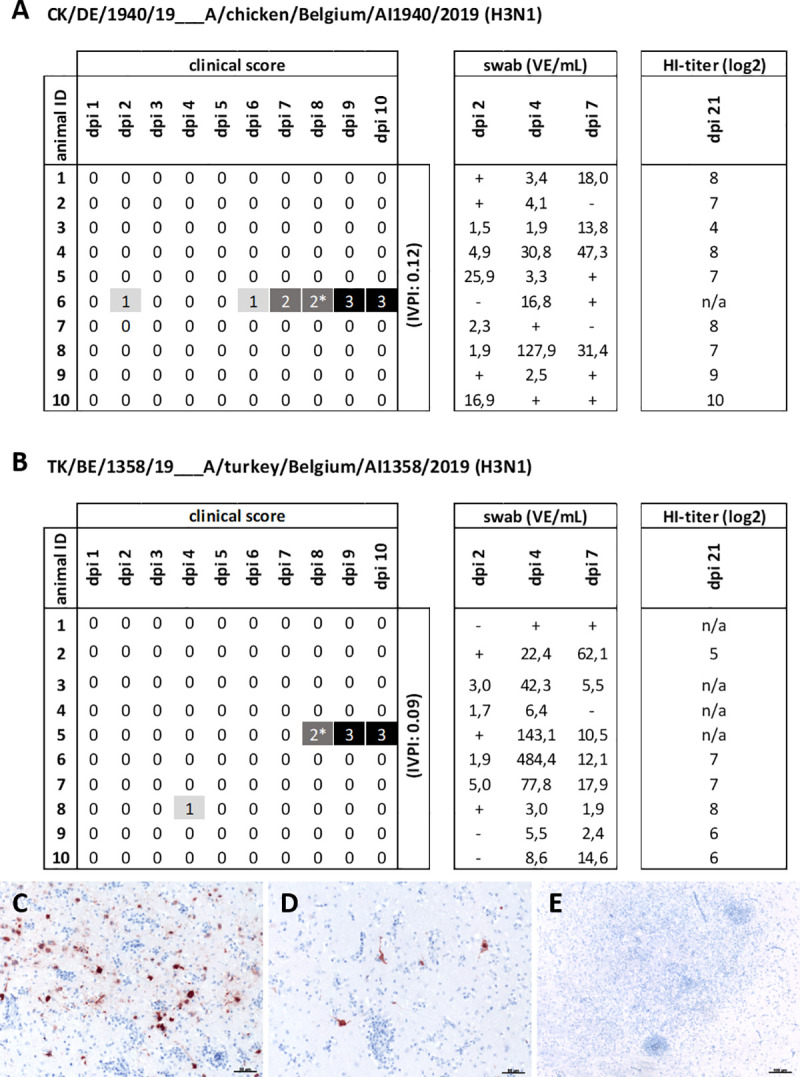
Intravenous infection of chickens with Belgian H3N1 isolates: Clinical course, HI-titers, and virus antigen detection. Chicken were infected intravenously (i.v.) with either (A) CK/DE/1940/19 or (B) DK/BE/1358/19 H3N1 isolate. The clinical status was monitored over a period of ten days post inoculation (dpi). Clinical status is recorded as healthy (0), birds showing slight (1) or severe disturbances of the general condition (2). In both groups, one animal showed neurological signs including torticollis and was euthanized, as indicated with asterisk. Subsequently they were recorded as dead (3). The IVPI is the mean score per bird per observation over the 10-day period (1 observation per bird per day). In addition, virus shedding was measured and presented here as infectious virus equivalents (VE) per ml, calculated on basis of cq values from combined oro-pharyngeal swabs and correlated to a standard curve of stock virus with known infectivity titer. VE below 1 are indicated with (+) and VE of 0 are indicated with (-). Finally, antibody HI-titers of sera were determined three weeks after infection. Viral matrix protein detection in the (C) brain stem and (D) cerebral cortex after i.v. infection with CK/BE/1940/19. (E) No antigen detection following i.v. TK/BE/1358/19 infection, despite significant inflammatory infiltration. Influenza virus matrix protein was detected by immunohistochemistry, using the ABC method, AEC as chromogen (red) and counterstained by haematoxylin (blue). Bar = 50 μm (C, D) and 100 μm (E).

Testing pathogenicity after i.v. inoculation of ten 6-weeks-old chickens per group demonstrated low overall pathogenicity for two Belgian AIV/H3N1 isolates (CK/BE/1940/19, TK/BE/1358/19), with an IVPI of 0.12 and 0.09, respectively (**Fig 1A and 1B**). Analyses of combined oral and rectal swab samples by RT-qPCR revealed a productive infection in all inoculated animals (**Fig 1A and 1B**). Already at 2 days post inoculation (dpi), the majority of animals shed virus, and all animals tested after three weeks seroconverted with homologous HI-titers (log_2_) of at least 4 (CK/BE/1940/19), respectively 5 (TK/BE/1358/19) (**Fig 1A and 1B**). While no overt clinical signs were recorded for the majority of inoculated birds (9/10), one animal in both groups developed severe neurological disorders at 7 and 8 dpi with torticollis (CK/BE/1940/19) or opisthotonos (TK/BE/1358/19) and had to be euthanized. Histopathology identified a subacute meningoencephalitis in both chickens with CNS disorders. Furthermore, immunohistochemistry (IHC) yielded viral antigen in neurons and glial cells of the cerebral cortex and brain stem in the chicken infected with CK/BE/1940/19 (**Fig 1C and 1D**). A focal myocarditis was present in the chicken suffering clinical disease after TK/BE/1358/19 infection, without demonstration of IAV antigen (**Fig 1E**). All other tissues investigated from both animals (lung, kidney, liver, pancreas, gastrointestinal tract, spleen and bursa) did not show infection-related alterations or IAV antigen. Examination of brain samples by RT-qPCR revealed AIV-RNA with cq values of 24.0 and 31.2 respectively for CK/BE/1940/19 and TK/BE/1358/19, indicating a higher virus load for the sample that also had an IAV antigen signal (CK/BE/1940/19). Taken together, we observed one chicken per group displaying severe neurological disorders, thus requiring euthanasia.

### H3N1 CK/BE/1940/19 replicates in neurons and exhibits endotheliotropism along with systemic spread in day-old chicks and *in ovo*

**Fig 2:**
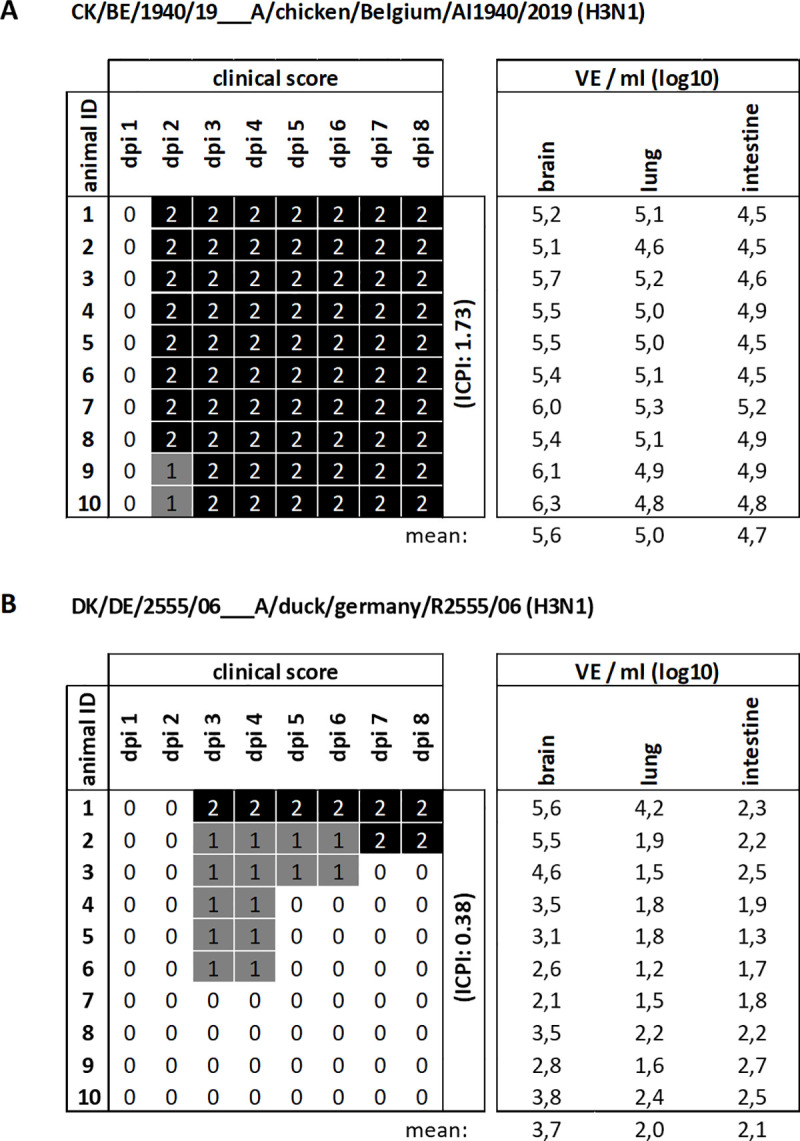
Intracerebral inoculation of chickens with H3N1 AIV: Clinical course and virus genome in organs. Day-old SPF chicks were inoculated intracerebrally either with (A) CK/BE/1940/19 (H3N1) or with (B) DK/DE/2555/06 (H3N1) virus isolates. The health status was scored daily over the period of eight days post inoculation (dpi), with (0) as healthy, (1) as diseased and (2) as dead. The ICPI is calculated as the mean score per bird per observation over the 8-day period (1 observation per bird per day). Organ samples were taken on day of death or 8 dpi and analyzed for virus genome load, presented here as infectious virus equivalents (VE)/ml, calculated by correlation of cq values to a standard curve of the respective virus stock with known titer.

The results of the IVPI demonstrated that both H3N1 isolates induce a productive infection and may spread to the CNS. To reveal the capacity of Belgian AIV H3N1 to replicate in neuronal tissue, we inoculated day-old specific-pathogen-free (SPF) chickens intracerebrally with CK/BE/1940/19 to monitor them clinically according to standard pathotyping procedures established for the Newcastle disease virus (NDV) [52]. Remarkably, all inoculated chicks suffered from peracute disease and were found dead at 2–3 dpi (**Fig 2A**). The resulting intracerebral pathogenicity index (ICPI) of 1.73 was equivalent to that of highly virulent NDV, capable of replication in the brain. Accordingly, high viral IAV load was detected by RT-qPCR in the brain, with systemic spread to the lung and intestine of all inoculated chicks (**Fig 2A**).

**Fig 3:**
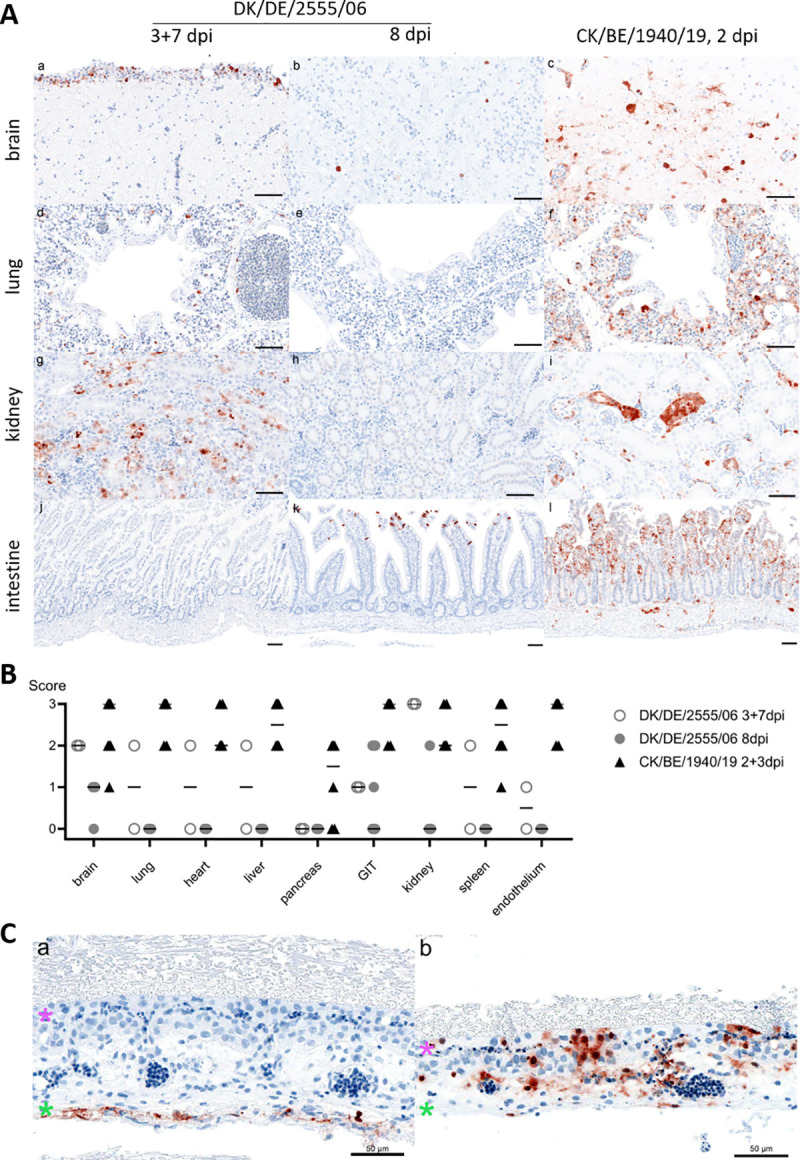
Tissue tropism of DK/DE/2555/06 and CK/BE/1940/19 following ICPI and inoculation of embryonated chicken eggs. (A) Representative images of IAV matrix protein (M1) detected in brain (a-c), lung (d-f), kidney (g-i) and intestine (j-l), following intracerebral inoculation of day-old chicks; time points indicate days post inoculation (dpi). ABC method, AEC chromogen (red), counterstain haematoxylin (blue). Bar = 50 μm. (B) Summary of semi-quantitative antigen detection in parenchymal cells of indicated organs. Dots represent individual animal tissue scores: 0 = no antigen, 1 = focal to oligofocal, 2 = multifocal, 3 = coalescing/diffuse. Bar indicates median, GIT = gastrointestinal tract. Details given in S1 Fig. (C) Immunohistochemical detection of IAV matrix protein (M1) in the CAM following allantoic inoculation of 10-day old embryonated SPF chicken eggs with either (a) DK/DE/2555/06 or (b) Ck/BE/1940/19 isolate. Indicated chorionic (pink asterisk) and allantoic epithelium (green asterisk). ABC method, AEC chromogen (red), counterstain haematoxylin (blue). Bar = 50 μm.

Histopathology and IHC confirmed a systemic virus spread, demonstrating abundant virus antigen-associated necrosis in all organs tested, including the brain, lung, heart, liver, pancreas, gastro-intestinal-tract (GIT), kidney, spleen and bursa (**Fig 3A and 3B**). Besides parenchymal cells, i.e. neurons and glial cells, pulmonary epithelium, hepatocytes, and others, endothelia throughout all examined organs were strikingly antigen positive (for detailed organ dissemination see **S1 Fig**).

By contrast, intracerebral inoculation with a non-related German H3N1 LPAIV DK/DE/2555/06 as control, resulted in only mild clinical manifestation in the majority of infected chicks, starting 3 dpi and only two animals died on day 3 and 7 (**Fig 2B**). The obtained ICPI (0.39) is equivalent to that of low virulent NDV. DK/DE/2555/06 spread to other organs, but interestingly, RNA loads determined by RT-qPCR in the lung and intestine, were at least at a 100-fold lower level compared to CK/BE/1940/19 (**Fig 2B**). Those results are in line with the histological findings: The most severely affected, i.e. bird which died on day 3 pi, showed viral antigen e.g. in glial cells, cardiomyocytes, renal tubules, pulmonary epithelium. In addition, sinusoid lining cells of the liver and single, but scarce endothelial cells of the lung and kidney were antigen positive (**Fig 3A and 3B**). The second animal which died on day 7 pi, showed antigen restricted to the site of inoculation and abundant signal in renal tubules, but not in lung or heart tissues and a complete lack of endothelial cell tropism. All remaining eight birds showed less abundant lesions and viral antigen, most consistently affecting the inoculation site (brain). Some birds showed antigen stain in peripheral organs including the kidney and intestine. Unlike to CK/BE/1940/19 infection, endotheliotropism was not evident in chickens euthanized at the end of the experiment at 8 dpi. Details including specifically affected cell types are given in **S1 Fig**.

These findings were corroborated by analyzing the spread of CK/BE/1940/19 and DK/DE/2555/06 in the CAM of embryonated SPF-chicken eggs. Common LPAIV as DK/DE/2555/06 typically replicate in the allantoic epithelium only, as demonstrated by immunohistochemistry (**Fig 3C**). In contrast, CK/BE/1940/19 antigen was found in all three CAM layers (chorionic epithelium, stroma, allantoic epithelium) and interestingly in the endothelium of CAM blood vessels (**Fig 3C**). Taken together, the LPAIV CK/BE/1940/19 (H3N1) displays notable systemic spread including neuroinvasion and endotheliotropism.

### The hemagglutinin endoproteolytic cleavage site (HACS) of CK/BE/1940/19 is not cleavable by furin

The results of both the IVPI and ICPI experiments of CK/BE/1940/19 indicated that, unlike typical LPAIVs, this virus was capable of productive replication beyond the epithelial borders of the respiratory and gastrointestinal tracts. Multistep replication associated with systemic spread is characteristically seen with *bona fide* HPAIV of subtypes H5 and H7 and requires HACS cleavage by the ubiquitous furin. In contrast, LPAIV HA is processed by trypsin-like serine peptidases, restricting replication mainly to the respiratory and digestive tract.

**Fig 4:**
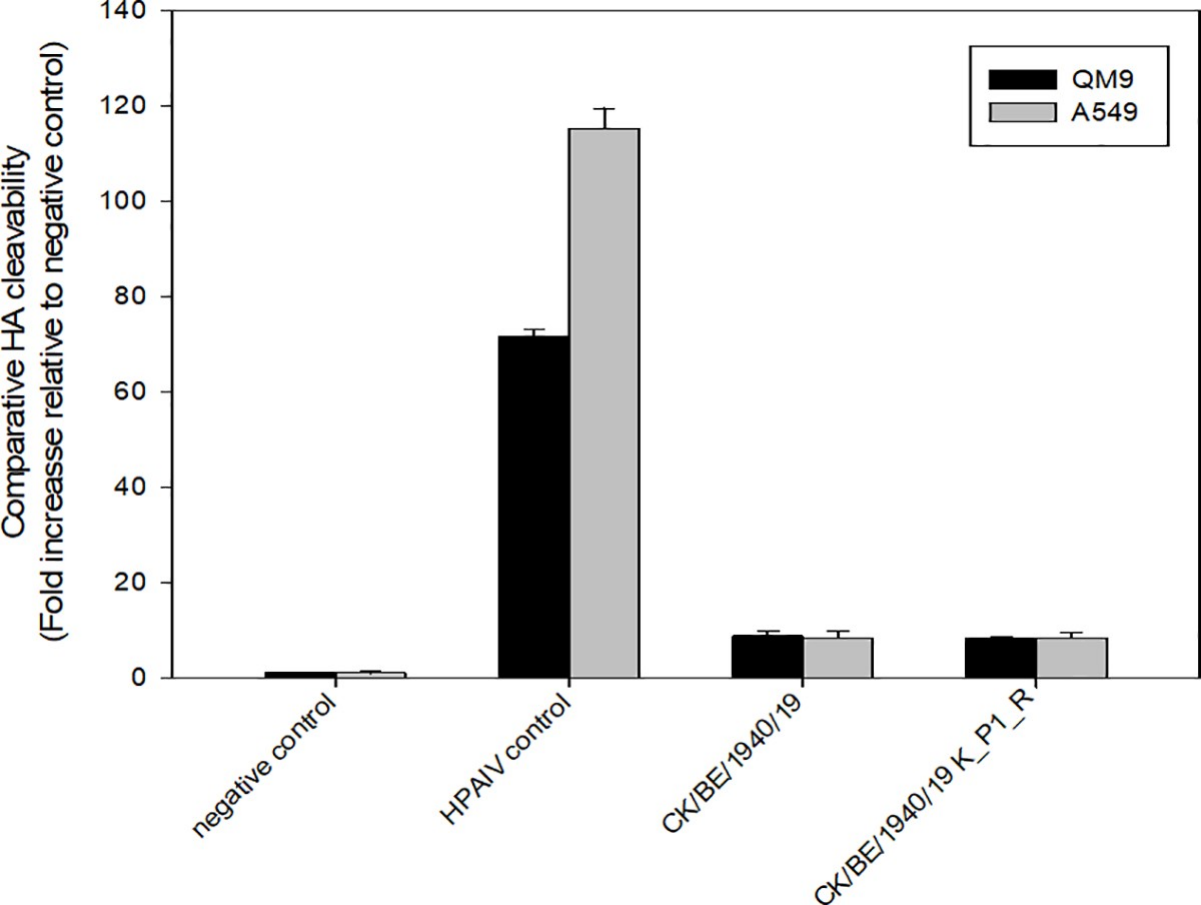
No furin cleavage of CK/BE/1940/19 (H3N1) HACS determined by luciferase reporter assay. Comparative cleavability of the hemagglutinin proteolytic cleavage site by furin or other intra-Golgi proteases, was determined by luciferase reporter assay in QM9 (avian) and A549 (human) cells. No signal was detected for the negative control, represented by a sequence with no HACS motif. For the HPAIV-derived polybasic HACS control a robust signal, more abundant in A549 cells, was generated. The CK/BE/1940/19 derived HACS, as well as a modified one with exchange of lysine at P1 against the more common arginine, did not induce signals indicating proteolytic processing.

To evaluate whether the HACS of H3N1 CK/BE/1940/19 is processed by intra-Golgi proteases, such as furin, the cleavability of CK/BE/1940/19 was analyzed by an *in vitro* luciferase reporter assay (qLUC) [53]. In this assay, the entire HACS including its vicinity (P14 to P5’ according to Tian et al. [54] is inserted between the reporter luciferase and a trans-Golgi network anchor sequence. Following transfection, furin-related intracellular cleavage is then signaled by activity of luciferase released to the supernatant. As positive control, the polybasic HACS of an H5 HPAIV (A/chicken/Bangladesh/AR134-c1/2016) was cloned into the same reporter construct and yielded extracellular luciferase activity after transfection indicating cleavage. However, the monobasic HACS of CK/BE/1940/19, in contrast, was not processed in the avian quail fibroblasts (QM9) or human pneumocyte (A549) cell lines transfected (**Fig 4**).

### Next-generation sequencing of CK/BE/1940/19 confirmed a stable monobasic HA cleavage site and a stable WSN-like loss of the N-glycosylation site N130 in N1

The full-genome sequence was determined by shot gun sequencing for the initial CK/BE/1940/19 virus stock, used for the infection experiments as well as for the virus in brain tissue from animal number ID 1 of the intracerebral inoculation experiment, which was found dead on 2 dpi. (accession number EPI_ISL_516830, www.gisaid.org). Metagenome analysis [55] did not reveal the presence of any other pathogen or infectious agent including NDV in both samples. Furthermore, the AIV H3N1 strain recovered after brain passage has maintained its monobasic HACS KQTK (P4-P1) and no minor variants of polybasic HACS variants were detected. In addition, no major change in mutation frequency on virus population level was detected by the variant analysis (**S1 Table**).

**Fig 5:**
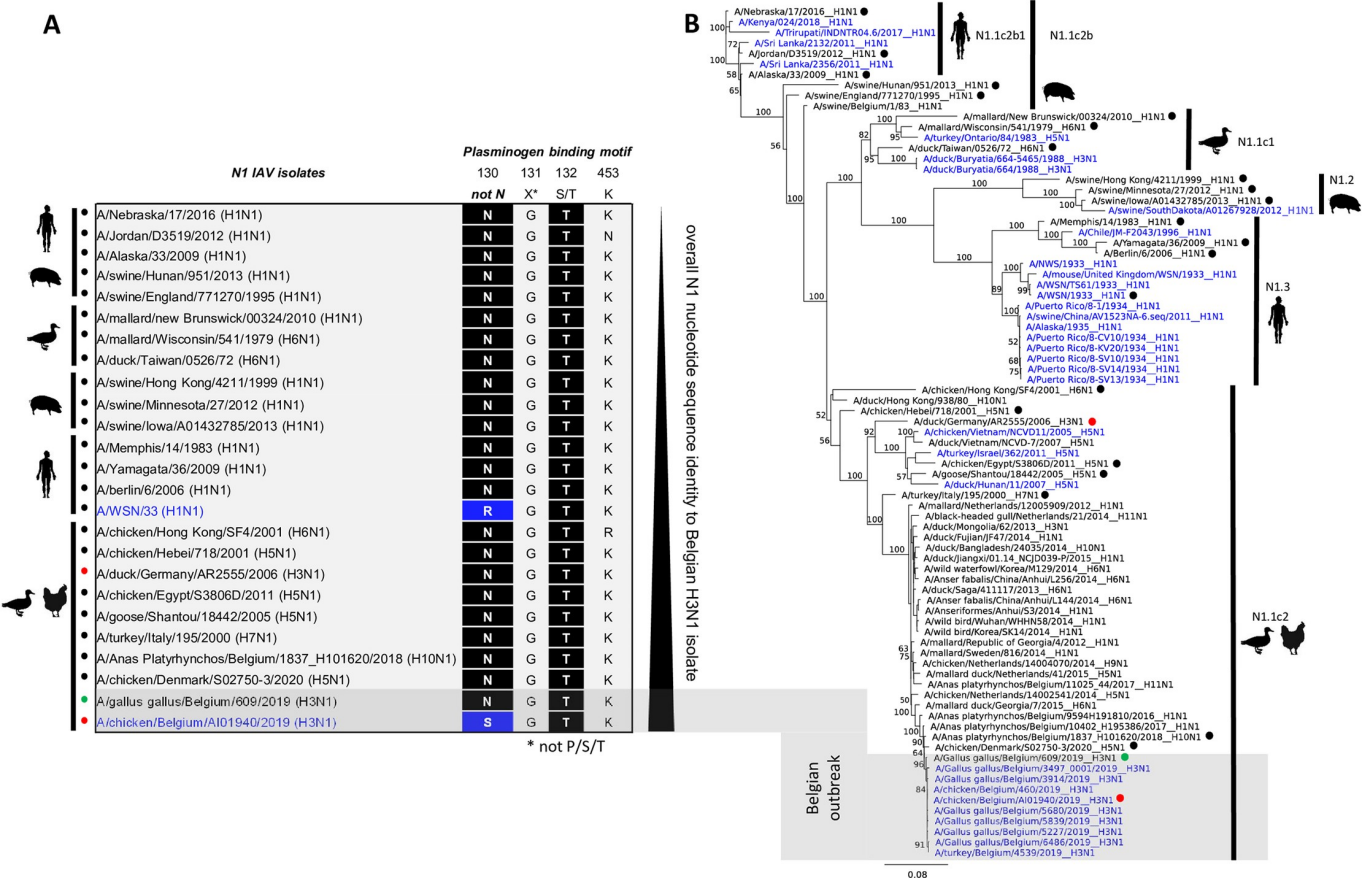
Sequence comparisons and phylogenetic analysis of the CK/BE/1940/19 neuraminidase (N1). (A) Amino acid alignment of positions described as responsible for neuraminidase mediated plasminogen binding. The loss of the Asn at position 130 and therefore the loss of the N-glycosylation site (blue isolate name), as described for WSN, has also been found in the CK/BE/1940/19 isolate but not in the comparative DK/DE/2555/06 isolate (red dots). First Belgian H3N1 isolate from January still has intact N-glycosylation site (green dot). Database research (www.fludb.de) including all full length N1-sequences without duplicates reveals only 24 of 11417 sequences to have no asparagine at this position. These 24 isolates (listed in detail in **S2 Table**) are shown in the (B) phylogenetic tree (calculated as a maximum likelihood tree using RAxML with a bootstrap value of 1000 cycles) together with the available N1-sequences from the Belgian H3N1 outbreak (n = 9) (both blue, except the Belgian January isolate) from 2019 and other representative N1 scaffold sequences (black).

Further sequence analyses identified a loss of the N-glycosylation site Asn130 (WSN numbering [56] (N130S) in the neuraminidase of CK/BE/1940/19 (**Fig 5A**); the substitution of this asparagine was previously shown to enable the NA to recruit plasminogen for proteolytic activation [40,43,44]. Database screening of 11,417 full gene sequences without duplicates of IAV NA N1 from different hosts revealed only 24 sequences with a similar loss of that site due to substitution of the asparagine by either an arginine (WSN), serine (CK/BE/1940/19) or others (**S2 Table**). The majority of identified strains are of human origin (H1N1, n = 16) including variants of the laboratory strains PR8 (n = 6) and WSN (n = 4). In addition, two strains of swine origin (H1N1) and six strains of avian origin representing H5N1 (n = 4) and H3N1 (n = 2) subtypes were found. It is interesting to note that eight out of nine isolates from the recent Belgian H3N1 outbreaks, for which published full-length N1-sequences are available, harbored the respective substitution (**Fig 5B**). Strikingly, the first Belgian H3N1 virus, which originated from the very first infected flock recognized in January 2019 (A/Gallus gallus/Belgium/609/2019), carries N130 in its NA, indicating no loss of the N-glycosylation site in this early phase. Phylogenetic analyses of the N1 segment revealed that the CK/BE/1940/19 clusters with other viruses from the Belgian H3N1 outbreak and are closely related to Belgian N1-sequences detected in AIV from mallards (*Anas platyrhynchos*) in 2018, 2017 and 2016 with nucleotide sequence identity of 99.34%, 99.26% and 98.84% respectively (www.blast.ncbi.nlm.nih.gov) (**Fig 5B**). A related N1-fragment with 99.03% nucleotide sequence identity surfaced again after the Belgian outbreak in an LPAIV H5N1 virus, detected on a chicken farm in Denmark in 2020 (A/chicken/Denmark/S02750-3/2020); but in this instance, the characterized N130 substitution was not present, strengthening the hypothesis that loss of the glycosylation site occurred during passage in captive poultry.

A monobasic HACS KQTR (P4-P1) was confirmed by full-genome analysis for the control wild duck strain DK/DE/2555/06 obtained during wild monitoring (accession number EPI_ISL_770631, www.gisaid.org). A nucleotide blast analysis of the DK/DE/2555/06 HA- and NA-segment sequences of show A/duck/Norway/1/2003 (H3N8) and A/common teal/Netherlands/2005 (H1N1) as the closest match with 98.53% and 99.51% identity respectively, corroborating the wild bird origin of DK/DE/2555/06. Compared to CK/BE/1940/19, the control wild ducks strain shares 89.58% identity and belongs to the same clade N1.1c (**Fig 5B**), but has an asparagine at position 130 (WSN numbering) in the N1, indicating an intact N-glycosylation site here. In summary, these data point to a high degree of conservation at position 130 of the N1 sequences studied, with rare, but repeated loss of that N-glycosylation site.

### The LPAIV CK/BE/1940/19 (H3N1) replicates in the absence of trypsin and depends on plasminogen for proteolytic activation

**Fig 6:**
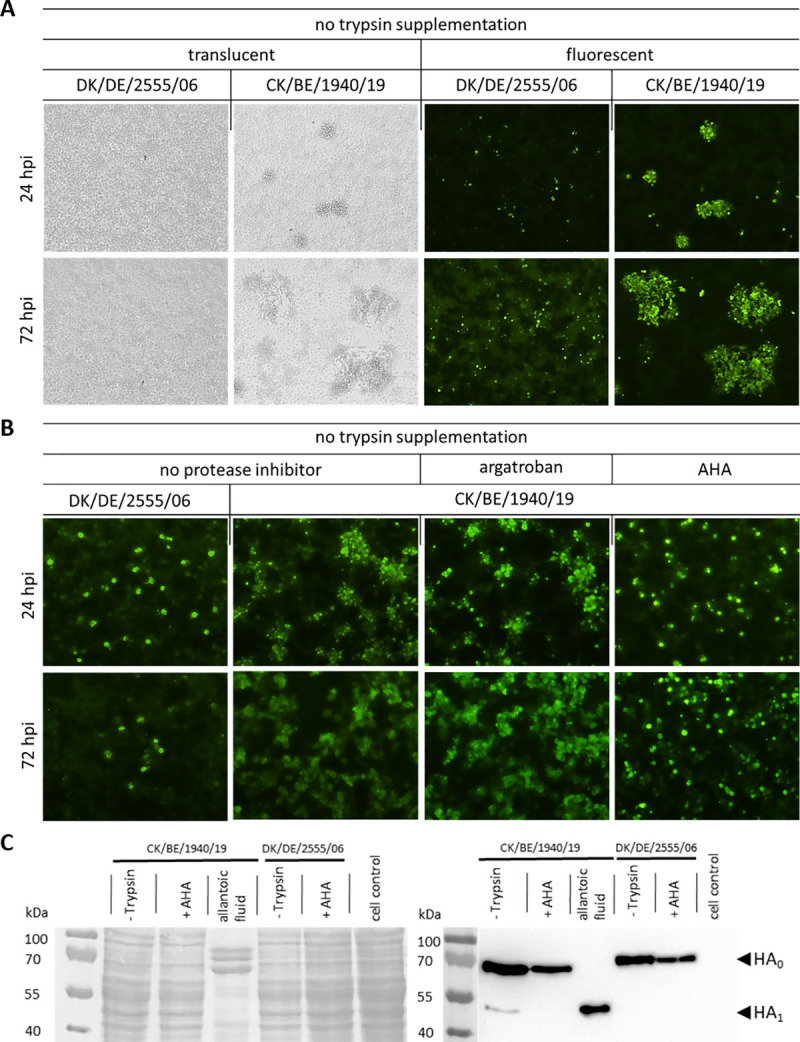
Trypsin-independent spread and HA_0_ cleavage of CK/BE/1940/19 is blocked by 6-aminohexanoic acid. (A) Immunofluorescent plaque assays on LMH cells were conducted with either DK/DE/2555/06 (H3N1) or CK/BE/1940/19 (H3N1) isolate. Cells were formalin fixated following incubation for 24 or 72 hours post inoculation (hpi). Cytopathogenic effect (CPE) was only observed for the CK/BE/1940/19 isolate by translucent light microscopy (left). Immunostaining confirmed IAV antigen is colocalized with visible CPE for the CK/BE/1940/10. Time-dependent increase of plaque size indicates ongoing virus spread. Only single individual cells stained positive with DK/DE/2555/06 and no increase in number of infected cells was detectable. (B) Trypsin-independent replication of CK/BE/1940/19 can be blocked by 6-aminohexanoic acid (AHA) supplementation in immunofluorescence plaque assays. While the LPAIV reference virus DK/DE/2555/06 only infect single cells without trypsin supplementation, the CK/BE/1940/19 H3N1 efficiently spread without trypsin and/or argatroban supplementation, a thrombin specific inhibitor. In contrast when cultivated with AHA, a plasmin specific inhibitor, CK/BE/1940/19 becomes restricted to single cell infection. (C) In vitro proteolytic cleavage of HA_0_ without trypsin-supplementation was prevented by AHA as shown by western blot. (left) Ponceau S stain proteins unspecific, confirming comparable protein loads of cell lysate at the membrane. (right) Chemiluminescent detection following incubation with H3 specific antibody (binding within HA_1_). There is a clear band at size of 70 kDa for the cell lysates of CK/BE/1940/19 and DK/DE/2555/06 inoculated cells, which representing the uncleaved HA_0_ protein. For CK/BE/1940/19, there is an additional band right below 55 kDa, representing the HA_1_, indicating proteolytic cleavage of HA_0_. This band do not appear when cultivated with AHA supplementation. In contrast, the allantoic fluid stock of CK/BE/1940/19 only, exhibits a strong HA_1_ specific signal.

To verify the suspected role of plasminogen/plasmin for HA_0_ activation of Belgian H3N1 viruses, we studied multicycle replication of CK/BE/1940/19 in immortalized chicken hepatocytes (LMH). Without external trypsin supplementation, LPAIV DK/DE/2555/06, harvested from embryonated eggs and thus already containing proteolytically activated infectious virus, initiated productive virus infections only in few single cells within 24 hours (detected by immunofluorescence, **Fig 6A**) but did not spread to surrounding cells during the subsequent 48 hours. This finding indicates that DK/DE/2555/06 is restricted to one-step replication in the absence of trypsin. In contrast, CK/BE/1940/19 produced visible foci in infected LMH cells after only 24 hpi; moreover, those foci further increased in size/cell numbers within the following 48 hours. Therefore, CK/BE/1940/19 was capable of multistep replication and cell-to-cell spread in the absence of trypsin (**Fig 6A**).

To investigate whether plasminogen recruitment is responsible for multicycle replication, specific protease inhibitors were applied to LMH cell cultures during virus inoculation in the absence of trypsin: Argatroban, a thrombin inhibitor [57] or AHA (6-aminohexanoic acid), a plasmin inhibitor [58]. After 72 hours of incubation, the CK/BE/1940/19-infected wells without inhibitor and those containing argatroban still showed visible cytopathic effects. In contrast, multicycle virus replication was inhibited in the presence of AHA (**S2 Fig**). Following fixation after 24 or 72 hpi and subsequent immunofluorescence analyses, the CK/BE/1940/19-inoculated cells in the presence of AHA revealed only isolated antigen-positive cells without further spread, similar to the non-related H3N1 LPAI isolate DK/DE/2555/06 when cultivated without trypsin supplementation (**Fig 6B**).

Consistent with the restricted spread of DK/DE/2555/06 in LMH cells without trypsin, western blot analysis using antibody specific for the N-terminal region of H3 HA, detected the non-cleaved HA_0_ precursor, only. In contrast, in supernatants from CK/BE/1940/19-infected LMH cell cultures, HA_0_ cleavage was indicated by occurrence of the HA_1_ fragment. However, in LMH cultures treated with AHA, only non-cleaved HA_0_ was detectable, demonstrating that plasmin is required for proteolytic HA activation of CK/BE/1940/19 (**Fig 6C**).

In conclusion, a plasmin inhibitor blocked trypsin-independent multicycle growth of CK/BE/1940/19 in LMH cells, indicating the requirement of plasmin for proteolytic activation.

## Discussion

In this study, we report that LPAIV H3N1 (Belgium 2019)—with a monobasic HACS—in initial screening experiments unexpectedly turned out to spread systemically and display neurotropism in some chickens. Replication in the brain of the two H3N1 isolates CK/BE/1940/19 and TK/BE/1358/19 studied was observed to some extent following intravenous inoculation. However, their low pathogenicity index was in agreement with the monobasic HACS, a marker of low pathogenicity in subtype H5 and H7 AIV [7]. Our results corroborate data of a previous study reporting an IVPI of 0.13 for an early virus isolate from the series of H3N1 outbreaks 2019 in Belgium [9]. However, for an LPAIV, we did not expect the sporadic CNS disorders observed in 10% of the inoculated birds, consistently associated with meningoencephalitis. Therefore, we further investigated the neurotropic virulence potential by intracerebral inoculation of the virus to determine the ICPI. This classical procedure is still the most sensitive assay to evaluate viral capacity for multicycle replication in the absence of extracellular trypsin-like proteases and by default to differentiate the NDV pathotypes [52]. For CK/BE/1940/19, a high ICPI of 1.73 was measured. Although few ICPI data are available for AIV, ICPI scores for LPAIV were reported to be ≤ 0.73 [59], while HPAI viruses had indices of at least 1.36 and higher [60,61]. In this respect, the LPAIV CK/BE/1940/19 behaves like an HPAIV. Accordingly, virus replication was prominent in all organs including the brain with abundant antigen labelling detected by immunohistochemistry. In contrast, another H3N1 wild bird isolate (DK/DE/2555/06) used as control for a common LPAIV had a considerably lower ICPI of 0.38. Besides expected focal to multifocal antigen detection at the inoculation site, systemic spread remained rare and markedly restricted. Our findings therefore support the general notion that the pathogenicity of IAV carrying a monobasic HACS is low and that neuropathogenicity is not a wide-spread characteristic in particular of H3N1 or H3NX subtypes [62–64].

Present results revealed furthermore that proteolytic HA activation of the Belgian H3N1 strain was independent from trypsin-like proteases, established as typical property of HPAIV due to their polybasic HACS [12]. Previously, for LPAIV a gain in pathogenicity and neurotropism could be obtained by sequential brain passages by enriching minor variants within the virus population accompanied by a stepwise accumulation of basic amino acids at the HACS [65]. For the HPAIV-like H3N1 strain CK/BE/1940/19, we excluded that possibility by NGS showing that both the inoculated and the brain-passaged virus isolated from the dead chick preserved the same monobasic HACS motif. In addition, NGS shot gun sequencing did not provide any evidence of further pathogens like NDV in either sample, excluding co-infections as causative for the observed neurovirulence.

Further analysis indicated trypsin-independent multicycle replication of CK/BE/1940/19 in cell culture and proteolytic cleavage of HA_0_. By contrast, the control virus DK/DE/2555/06, whose HA_0_ was not cleaved, did not show any trypsin-independent spread. In addition, involvement of intracellular proteases like furin could be excluded by an *in vitro* luciferase assay. Argatroban, a thrombin-specific inhibitor, however, did not have an effect on multicycle replication, excluding involvement of thrombin in the trypsin-independent replication, hence thrombin-mediated cleavage of the monobasic HACS has been described for Eurasian LPAIV H7N6 [57]. Sequence analysis corroborate this results, since a thrombin-cleavable HACS should include the motif GR or PR/K (P2-P1) for successful cleavage, not present in the studied Belgian H3N1 virus isolate [66,67]. In agreement with our hypothesis of WSN-like neuraminidase-mediated plasminogen recruiting for HA_0_ cleavage, leading to trypsin-independent growth and neurovirulence, a competitive plasmin inhibitor (AHA) [58,68] was able to block multicycle replication *in vitro*, limiting infection to single cells and prevented proteolytic cleavage of HA_0_. Hence, our findings are in line with properties described for the A/WSN/33 virus that exhibits the same N-glycosylation site loss in the NA at position 130, enabling plasminogen binding [62–64]. In addition, the identified monobasic HACS KQTK (P4-P1) of CK/BE/1940/19 contains nearly an optimal motif for plasmin, consisting of KX(W/Y/F)K (P4-P1), while the threonine is not part of that optimal motif but was classified as sixth best suitable amino acid at P2 [66]. Therefore, the KQTK motif of CK/BE/1940/19 does not resemble the common H3 HACS consensus sequence, described as KQTR (P4-P1), and in addition differs from that of the A/WSN/33, IQYR (P4-P1) [69]. In case of WSN/33 it has been shown, that the amino acid substitution at P2 (Y382S) drastically reduces the cleavability by plasmin correlating with lower virus titers in mouse brain but not in lung, suggesting the importance of a suitable HACS for plasmin utilization in addition to the efficient plasminogen recruiting [69]. It is tempting to speculate that the rare exchange of R to K in P1 increases the susceptibility of the CK/BE/1940/19 HA to plasmin cleavage and that a common minimal H3 plasmin motif could be KXXK (P4-P1), yet remaining to be tested. Here, already two residues at P4 and P1 from the three non-variable positions P4, P2, and P1 of the previously mentioned optimal plasmin cleavage motif [66] are identical.

With regards to neurovirulence, the most striking effects were derived from mutations within the NA-spike protein of the mouse-adapted H1N1 strain WSN. The loss of a glycosylation site due to substitution of N130R in the NA of WSN enabled plasminogen binding at the C-terminal lysine residue of the N1 protein [40,43,44]. In accordance, the reconstitution of that glycosylation site (WSN-NA R130N) leads to loss of neurovirulence, trypsin-independent replication in cell culture and the ability to utilize plasminogen [43]. Our sequence analysis revealed that the further studied H3N1 isolate (CK/BE/1940/19) had also lost this N-glycosylation site. The zymogen plasminogen is ubiquitously prevalent, including in neurons, [48,70] and needs to be proteolytically activated to plasmin by its major activators urokinase-type plasminogen activator (uPA) or the tissue-type plasminogen activator (tPA) [71,72]. These activators are also nearly ubiquitously present in all organs, with highest activity in lung, uterus, brain, kidney and adrenal gland [73]. Together, such a distribution of plasminogen and its major activators would explain the ability of CK/BE/1940/19 to replicate systemically including neuronal tissues despite a monobasic HACS. Beside that the most information about plasminogen distribution and function are derived from mammals, the plasminogen system is mostly conserved in chordates [74] and there are strong indications that most of them are also basically applicable for avian species, here chickens [75–77]. In addition, endothelial cells are highly active plasminogen producers, secreting plasminogen but also the membrane-bound uPA-receptor that mediates plasminogen and uPA colocalization on the membrane surface [78–80]. This deduced plasminogen tissue distribution could explain the observed endothelial cell tropism for the CK/BE/1940/19 H3N1 isolate. Furthermore, endothelial cell tropism has been described as crucial for systemic spread of H5N1 HPAIV in mammals [81,82], but with some species-specific differences in avian hosts [83]. Hence the blood-brain barrier itself is composed of closely connected endothelial cells [84], this could enable the virus to enter (or leave in case of intracerebral inoculation) the brain by infection of the endothelial cells of the blood-brain barrier giving a potential link between the observed endothelial tropism, neurotropism and systemic spread.

Beyond the clinical features of the AIV H3N1 outbreak in Belgium, the found essential contribution of the NA N1 highlights the NA N130S mutation, recognized in the laboratory strain WSN [40,43,44], to be a general virulence determinant. According to its very low frequency in published sequences, this mutation seems to be a rather exceptional event, as our database analysis showed that an asparagine at position 130, crucial for the N-glycosylation, was strongly conserved in 99.8% of 11,417 available N1 sequences. Strikingly, the exchange WSN-NA N130S was not detected in the N1 sequence derived from the index farm of the first Belgian H3N1 outbreak in January 2019, but in all the eight N1 sequences later available from this outbreak. This first sequence probably reflects the ancestor sequence of circulating N1 in wild birds, since close N1 blast hits are derived from duck strains in Belgium of the years 2018, 2017 and 2016. In addition, the closest related sequence originated from an H5N1 LPAIV from a Danish poultry farm in 2020, several months after the Belgian outbreak was extinguished [85]. It is therefore likely that the loss of NA N1 N130 originated during further spread among gallinaceous poultry along the ongoing epizootic. Relatives of this N1 are however still circulating, as indicated by the sequence from Denmark 2020 (A/chicken/Denmark/S02750-3/2020(H5N1)), probably also bearing the potential to lose this N-glycosylation site by mutation again. The NA subtype N1 is compatible with a wide range of HA subtypes and in accordance phylogenetically related N1 subtypes were found in several reassortants like H1N1, H5N1, and H10N1, infecting both animal and human hosts [86]. Moreover, the emergence of the Belgian H3N1 NA mutant shows that pathogenicity in IAV is a polygenic trait, often affecting genetic loci other than such as the HACS or receptor binding regions. Therefore, long-lasting transmissions and replication of AIV in very large populations of gallinaceous poultry may provide a huge melting pot for emergence of novel virus mutants with unknown virulence potential. Because of those dynamic mechanisms, future surveillance programs of poultry and wild bird populations should not only be focused on the H5, H7 or H9 AIV, but also include H1 and H3 strains. Even if conjunctival swabs taken from farmers of the H3N1-positive holdings were tested negative and no zoonotic transmission was reported yet [9], such novel N1 mutants may serve in reassortment events as donators of gene segments which mediate a gain in pathogenicity through increased neurovirulence and endotheliotropism in human-adapted IAV.

## Material and methods

### Ethics statement

All animal experiments were conducted in biosafety level 3 containment facilities at the FLI and were carried out in accordance with the German Animal Welfare Act, approved by the Committee on the Ethics of Animal Experiments of the Federal State of Mecklenburg-Western Pomerania (registration and approval number LALLF MV/TSD/7221.3-2-009/19).

### Viruses and cells

Three viruses of subtype H3N1 were selected for comparison: A/chicken/Belgium/AR1940/2019 (H3N1) in the following designated as CK/BE/1940/19 and A/turkey/Belgium/AI1358/2019 (H3N1) in the following designated as TK/BE/1358/19, were derived from serious clinical outbreaks in poultry holdings in Belgium, 2019. The isolate A/duck/Germany/AR2555/2006 (H3N1) (DK/DE/2555/06) was obtained from routine diagnostic poultry samples from domestic ducks in Germany in 2006. All isolates were used following the third egg passage.

All cell cultures were cultivated at 37°C and 5% CO_2_ concentration. Japanese quail fibroblasts [QM9 (CCLV-RIE 466: CVCL-0I49)], and a human pneumocyte-II lineage [A549 (ATCC) CCL-185] were cultured with specific cell culture medium containing 5,32 g/L Ham’s F-12 Nutrient Mixture (Thermo Fisher Scientific, Waltham MA, USA), 8,80 g/L Iscove’s Modified Dulbecco’s Medium (IMDM) (Thermo Fisher Scientific, Waltham MA, USA), 2,45 g/L NaHCO_3_ (Carl Roth, Karlsruhe, Germany) and 10% fetal calf serum (FCS). Immortalized chicken hepatocytes [LMH (ATCC (CRL-2117), 1997] were cultivated with 9,9 g/L Dulbecco’s Modified Eagle Medium (DMEM) (Thermo Fisher Scientific), 0,86 g/L NaHCO_3_ (Carl Roth) and 10% FCS.

We used embryonated chicken eggs (ECE) to propagate the viruses and generate the virus stock, as well as for immunohistochemical examination. Specific-pathogen-free (SPF) ECE were received from VALO BioMedia GmbH, Osterholz-Scharmbeck, Germany, and incubated for ten days. 200 μl of a 1:1000 dilution of CK/BE/1940/19 and DK/DE/2555/06 primary egg culture was inoculated in the allantoic cavity. Allantoic fluid was harvested after 72 hours, centrifuged at 2000 rcf to get rid of cell debris and aliquoted before storage at– 80°C. The remaining egg membranes and the embryo were fixated with 4% formalin and subsequently used for IHC analyses.

### Animal experiments

White leghorn chickens (*Gallus gallus* var *domesticus*) hatched from SPF eggs (VALO BioMedia GmbH, Osterholz-Scharmbeck, Germany) at FLI and were kept in free range on the ground of separate stables.

We ran an intravenous pathogenicity index (IVPI) testing according to the World Organisation for Animal Health (OIE) routine procedures (2006/437/EC) [87], with minimal adaptations. Ten AIV–seronegative chicken each received 100 μl of a 1/10 virus/saline dilution of subtype H3N1 CK/BE/1940/19 or TK/BE/1358/19. For each isolate ten animals were intravenously infected. In addition to clinical scoring, virus shedding was examined by sampling combined oral-cloacal swabs at days 2, 4 and 7. Swabs were placed in 1 mL DMEM supplemented with Enrofloxacin (Bayer, Leverkusen, Germany). The animals were clinical scored as “healthy = 0”, “sick = 1”, “severely sick = 2”, “dead = 3” daily over a period of ten days post inoculation (10 dpi), as shown in **Fig 1**. The IVPI is the mean score per bird per observation over the 10-day period (1 observation per bird per day). Finally, surviving animals were kept until 21 dpi with the intention to evaluate the serological response.

The intracerebral pathogenicity index (ICPI) was conducted according to the standard procedure for Newcastle Disease (ND) [52] for the CK/BE/1940/19 and the DK/DE/2555/06 isolate, using 50 μl of a 1/10 virus stock in saline dilution. Briefly, after disinfection of the skin with parting the feathers chicks were inoculated with an insulin syringe using a 26G needle (0,45 x 12 mm), applying the virus paramedian into the right forebrain. The animals were clinical scored as “healthy = 0”, “sick = 1”, “dead = 2” over a period of eight days post inoculation (8 dpi), as shown in **Fig 2**. The ICPI is calculated as the mean score per bird per observation over the 8-day period (1 observation per bird per day). Brain, lung and intestinal samples of chicks succumbing to the infection were taken for PCR analysis, while an advanced organ panel used for pathology and immunohistochemistry as described below.

### Real-time reverse transcription polymerase chain reaction (real-time RT-qPCR)

Viral RNA from swab and organ samples were extracted using the NucleoMag VETkit (Macherey-Nagel, Düren, Germany) in combination with a Biosprint 96 platform (Qiagen, Hilden, Germany). IAV RNA was semi-quantified by real-time reverse transcription polymerase chain reaction (RT-qPCR) using a target sequence for amplification within gene segment 7 adapted from [88]. All RT-qPCR reactions were performed in 12.5 μl volumes, using the AgPath-ID One-Step RT-PCR kit (Ambion, Foster City, CA, USA) on a CFX96 thermocycler machine (Bio-Rad Laboratories, Hercules, USA) with 42 cycles. RT-qPCR Cq threshold was set at 39.9. The cq values were correlated to TCID_50_/ml infectious titers using a titration of the CK/BE/1940/19 or, respectively, the DK/DE/2555/06 virus stock in MDCK cells to generate a standard curve. Therefore, the cq value (y) of the sample was used to calculate the virus equivalent (VE) titer (x) by applying the determined regression formula y = -1,302ln(x) + 35,703 (DK/DE/2555/06), respectively y = -1,509ln(x) + 35,03 (CK/BE/1940/19) (S3–S5 Figs).

### Next-generation sequencing

RNA extraction of the influenza positive H3N1 sample was conducted with the QIAamp Viral RNA Mini KIT (Qiagen, Hilden, Germany) and Trizol LS (Thermo Fisher Scientific, Waltham, USA) according to the manufacturer’s instructions. A universal influenza primer pair designed to bind to the conserved termini [89] were employed in combination with the Invitrogen Superscript III One-Step RT-PCR and Platinum Taq (Thermo Fisher Scientific) to simultaneously amplify all genome segments. Subsequently, the amplicons were purified with AMPure XP Magnetic Beads (Beckman Coulter, Fullerton, USA).

Sequencing of the purified RT-PCR amplicons was conducted on the IonTorrent platform (Thermo Fisher Scientific) as previously described [90]. In short, the sample was mechanically fragmented (500 bp size) on a Covaris M220 Ultrasonicator (Covaris Ltd., Brighton, UK). Library preparation was performed with the GeneRead DNA L Core Kit (Qiagen) and Xpress Barcode Adapters (Qiagen) to achieve an end-repaired and barcoded library. After a succeeding size-selection step with AMPure XP Magnetic Beads (Beckman Coulter), the library underwent a quality check on an Agilient Bioanalyzer 2100 (Agilent Technologies, Böblingen, Germany) and was quantized with the KAPA Library Quantification Kit (Roche, Mannheim, Germany). Sequencing was performed on the IonTorrent S5XL (Thermo Fisher Scientific) in combination with the Ion OneTouch 2 System (Thermo Fisher Scientific).

The raw sequencing data was screened for adapter and primer contamination before quality trimming. Consensus sequence production was performed by using the Geneious Software Suite (v11.1.5; Biomatters, Auckland, New Zealand) in a map-to-reference approach with Bowtie2 (v2.3.0; preset “Highest sensitivity”) [91].

### Phylogenetic and database analysis

Database research regarding the absence of an asparagine at position 130 (WSN numbering) was conducted on all available full length N1 sequences without duplicates on the influenza research database (IRD) (www.fludb.org). In order to determine the closest relatives on gene segment level of the CK/BE/1940/19 N1 gene segment a nucleotide blast search was conducted using the Global Initiative on Sharing All Influenza Data (GISAID) database (www.gisaid.org). The phylogenetic tree was calculated as a maximum likelihood tree using RAxML with a bootstrap value of 1000 cycles. At least three scaffold N1 sequences were chosen to represent N1 lineages and subordinate lineages as they were described by Zhuang et al. 2019 [86]. The results of the IRD and the first 50 blast results, with bootstrap values of 50 or higher were included into the phylogenetic analysis.

### Plaque and immunofluorescence assays

LMH cells were seeded into 48 well plates (Corning Costar, Corning, NY, USA). After 24 hours, they were inoculated with 100 μl of a 10^−3^ dilution of the respective viruses, that contain 10^7,38^ TCID_50_/ml (DK/DE/2555/06) and 10^6,75^ TCID_50_/ml (CK/BE/1940/19) respectively. Following 1-hour incubation at 37°C and gentle swirling, we removed the supernatant and washed the cells three times with saline solution. Now the cells were covered with 0.8% carboxymethyl cellulose (CMC) in DMEM with 10% FCS. After 24 and 72 hours of incubation at 37°C, cells were fixated with 4% formalin. For subsequent immune staining, cells were permeabilized with 1% Triton-100 in phosphate-buffered saline (PBS) for 30 min, followed by incubation with blocking buffer (1% bovine serum in PBS) for 30 min at room temperature. Blocking buffer was removed and primary antibody (Ab), diluted 1:50 in blocking buffer was added. Here we used polyclonal chicken sera specific against A/chicken/Belgium/AI1940/2019 (H3N1) (derived from the IVPI experiment) or, respectively, against A/duck/Germany/R2555/06 (H3N1) (hyperimmune serum raised against β-propiolacton inactivated full virus). After 1 hour of incubation we washed the cells three times with washing buffer (0,025% Tween-20/PBS), before adding the fluorescein isothiocyanate (FITC) labeled secondary Ab (goat-anti-chicken-IgY-FITC (OriGene Technologies GmbH, Maryland, USA)), diluted 1:400 in blocking buffer. Following 1-hour incubation, the cells were washed three times with washing buffer and finally 20% glycerol in PBS was added for evaluation on a fluorescence microscope.

### Protease-inhibitor assay

LMH cells were seeded into 96 well plates (Corning Costar, Corning, NY, USA) with DMEM containing 10% fetal calf serum. After 24 hours, medium was removed and virus inoculum (100 μl medium with 10^3^ TCID_50_/well) was added. The inoculum contained either no additional proteases inhibitor, 6-aminohexanoic acid (AHA) (Merck KGaA, Darmstadt, Germany) in a final concentration of 10 mg/ml or argatroban (Santa Cruz Biotechnology Inc, Dellas, TX, USA) in a final concentration of 50 μg/ml. Cells were incubated for 1 hour at 37°C. After this the infection medium was removed and 200 μl DMEM without FCS, but containing the respective inhibitor (none, AHA or argatroban) at the same concentration was added. Finally, cells were covered with CMC in a final concentration of 0.6% CMC. From that step, the following procedure was continued as described for the plaque immunofluorescence assay.

### Endoproteolytic cleavage sensitivity measured by a luciferase assay

Plasmids were constructed based on the pSELECT-N-Lucia system (InvivoGen, USA) as described previously [53]: A secretable luciferase reporter (qLUC) was linked to a trans-Golgi network (TGN) anchor sequence. As a linker between these two sequences, the hemagglutinin cleavage site (HACS) region of A/chicken/Belgium/AI1940/19 [H3N1] (LATGMRNVPEKQTK*GLFGA) was introduced in frame. As a positive control, a construct expressing a polybasic HACS sequence (LATGLRNSPQRERRRKR*GLFGA) derived from the HPAIV subtype H5N1 (A/chicken/Bangladesh/AR134-c1/2016 [H5N1, clade 2.3.2.1a]) was used. Another construct lacking any HACS served as a negative control. Sequence identity was confirmed by Sanger sequencing, and plasmids were purified by the QIAfilter Plasmid Maxi kit (Qiagen) before transfection for protein expression. qLUC is only released and secreted into the culture medium if endoproteolytic processing has been accomplished. Japanese quail fibroblasts [QM9 (CCLV-RIE 466: CVCL-0I49)], and a human pneumocyte-II lineage [A549 (ATCC) CCL-185] were transfected and chemoluminescence activity in collected supernatants was determined using the QUANTI-Luc assay (InvivoGen, USA) according to the previously described protocol.

### Hemagglutination inhibition assay

Productive infection was evaluated by detection of antibodies by the hemagglutination inhibition assay (HI-assay). Viral antigen (CK/BE/1940/19) was adjusted to four HA units. Twofold dilution series of sera from log_2_ 1 until log_2_ 11 were prepared with saline in 96-well plates (Greiner AG, Kremsmünster, Austria), resulting in 25 μl volume for each. 25 μl adjusted viral antigen was added to each dilution and incubated for 45 min at room temperature. Finally, 25 μl of 1% chicken erythrocytes suspension were added. Following 30 min of incubation at room temperature, plates were read after perpendicularly tilting the plates. Inhibition of hemagglutination indicates presence of specific antibodies. Cross-reactivity between CK/BE/1940/19 and DK/DE/2555/06 is given in S6 Fig.

### Western blot

LMH cells were grown to confluence in six-well cell culture plates (Corning Costar, Corning, NY, USA) TC-Treated Multiple Well Plates). Cells were inoculated with the respective viruses (MOI of 0.035) of CK/BE/1940/19 or DK/DE/2555/06). After incubation for 1 hour at 37°C, the infection medium was removed and cells were washed one time with DMEM and fresh medium was added. Cultures were grown in the absence of trypsin and one set each of wells received 6-aminohexanoic acid (20 mg/mL) supplementation in 2 mL DMEM without FCS. The whole cell layer was harvested after 24 hours incubation at 37°C, by using a cell scraper. Following short centrifugation for 1 minute at 20000 rcf, pellet was taken up in 100 μl Triton X100 lysis buffer (0.02 M disodium phosphate, 2 mM EDTA, 0.15 M NaCl, 1% (v/v) Triton X-100, pH 7.6). Samples were mixed with reducing SDS-polyacrylamide gel electrophoresis (PAGE) sample buffer (ROTI Load 1 reducing 4x; Carl Roth, Karlsruhe, Germany) in a ratio of 1:4 and heated at 99°C for 3 minutes. Per lane 15 μl were applied to a 10% acrylamide gel., that was run for 50 minutes at constant 200 V. Gel was washed in standard transfer buffer and blotted to a nitrocellulose membrane using Trans-Blot SD Semi-Dry Electrophoretic Transfer Cell (Bio-Rad, Hercules, CA, USA) at 15 V constant for 75 minutes. Protein transfer was verified by incubation of the membrane with Ponceau S stain (0.1% (w/v) Ponceau S (Carl Roth, Karlsruhe, Germany) in 5% acetic acid) for 5 minutes and subsequent flushing with water. Membrane then was blocked with blocking buffer (1% milk powder, Carl Roth, Karlsruhe, Germany, in PBS-Tween) for 1 hour. A polyclonal H3 specific IgG raised in rabbit against the N-terminal region of A/TW/3446/02 (H3N2) (Cat No. GTX127363, GeneTex, Irvine, CA, USA) was used as primary antibody, diluted 1:3000 in blocking buffer and incubated over night at 4°C. After washing with PBS-T, the secondary antibody, an anti-rabbit IgG, conjugated with peroxidase (A0545 (SKU), Sigma-Aldrich, St. Louis, MO, USA) in a dilution of 1:5000, was applied for 1 hour at room temperature. Following an additional washing step SuperSignal West Pico Chemilumineszenz-Substrat (Thermo Fisher Scientific, Waltham, MA, USA) peroxidase substrate was applied and detection was conducted by the Bio-Rad ChemiDoc XRS+ and Image Lab Software.

### Pathology and immunohistochemistry

Following intracerebral administration, we performed full autopsy 2–7 days after DK/DE/2555/06 infection or 2–3 after CK/BE/1940/19, respectively (n = 20 in total). Following intravenous CK/BE/1940/19 or TK/BE/1358/19 IVPI- administration, one chicken each was killed 8 days after infection. We collected brain, lung, heart, kidney, liver, pancreas, gastro-intestinal tract, spleen, and bursa, and fixed them in 10% neutral-buffered formalin. Additionally, the chorioallantoic membrane was collected and fixed after infection of embryonated SPF chicken eggs with either CK/BE/1940/19 (n = 1) or DK/DE/2555/06 (n = 1). The tissues were trimmed for paraffin embedding and 2-3-μm-thick sections were stained with hematoxylin and eosin (HE). The severity of necrotizing inflammation was scored on an ordinal 0 to 3 scale: 0 = no change; 1 = mild, focal; 2 = moderate, multifocal, and 3 = severe, diffuse necrosis. Immunohistochemistry was performed for viral antigen detection using a primary antibody against the M protein of IAV (ATCC clone HB-64) [92]. The extent of viral antigen labelling was scored on a 0 to 3 scoring scale: 0 = no antigen, 1 = focal to oligofocal, 2 = multifocal, 3 = coalescing/diffuse.

## Supporting information

S1 Table Deep-sequencing analysis of the CK/BE/1940/19 (H3N1) inoculum prior to ICPI and from a brain sample 2 dpi (animal 1). (JPG)

S2 Table IAV isolates identified by N1-sequence analysis (*www*.*fludb*.*org*) for lack of N-glycosylation site at position 130. (JPG)

S1 Fig IAV tissue tropism after intracerebral infection of chicks with DK/DE/2555/06 (H3N1) or CK/BE/1940/19 (H3N1). Semi quantitative antigen distribution in the brain (A), lung (B), heart, liver, pancreas and kidney (C), spleen, bursa, peripheral nervous system, smooth muscle cells and endothelium within all tissue (D), gastrointestinal-tract (E), endothelium in selected tissues (F). Dots represent individual animal tissue scores: 0 = no antigen, 1 = focal to oligofocal, 2 = multifocal, 3 = coalescing/diffuse. Bar indicates median, ep. = epithelium, PNS = peripheral nervous system, GIT = gastrointestinal tract.
(JPG)

S2 Fig Trypsin-independent cytopathogenic effect of CK/BE/1940/19 (H3N1) in LMH cell cultures can be blocked by AHA. While the LPAIV reference virus DK/DE/2555/06 did not cause visible CPE without trypsin supplementation, the CK/BE/1940/19 H3N1 induced pronounced CPE with complete lysis of the cell layer. CK/BE/1940/19-induced CPE is totally blocked when cultivated in the presence of 6-aminohexanoic acid (AHA), but not with argatroban supplementation.
(JPG)

S3 Fig Standard RT-qPCR curve used for infectious virus equivalent (VE) calculation of (A) DK/DE/2555/06 or (B) CK/BE/1940/19 samples.
(JPG)

S4 Fig Calculation of the infectious virus equivalent (VE) calculation of the IVPI samples using the cq values and the standard curve derived formula. (JPG)

S5 Fig Calculation of the infectious virus equivalent (VE) calculation of the ICPI samples using the cq values and the standard curve derived formula. (JPG)

S6 Fig Cross reactivity of sera from H3N1/BE infected chicken. Minor antigenic difference between homologous Belgium strain (CK/BE/1940/19) or heterologous wild bird AIV H3N1 (DK/DE/R2555/06) was observed by HI when sera from chicken inoculated for the IVPI experiment with either CK/BE/1949/19 (n = 9, red) or TK/BE/1358/19 (n = 7, green), obtained 21 weeks after infection. Beside individual values (□) and arithmetic means (○) of the groups, boxblots are representing results of combined results of both groups. No statistically significant difference were evident between infected groups (P = 0,142, t-test, Sigma Plot, Systat Software). A homologues reference serum to DK/DE/R2555/06 (◊) is derived from an immunized chicken and is producing reciprocal results of HI titer (log2) of 7 with homologous DK/DE/R2555/06 antigen vs. HI titer (log2) of 6 with heterologous CK/BE/1940/19.
(JPG)
