# Supplementary figures and images for: Neuraminidase-associated plasminogen recruitment enables systemic spread of natural avian Influenza viruses H3N1
Source: PLoS Pathog. 2021 Apr 23;17(4):e1009490. doi: 10.1371/journal.ppat.1009490 (PMC8118554; doi:10.1371/journal.ppat.1009490)

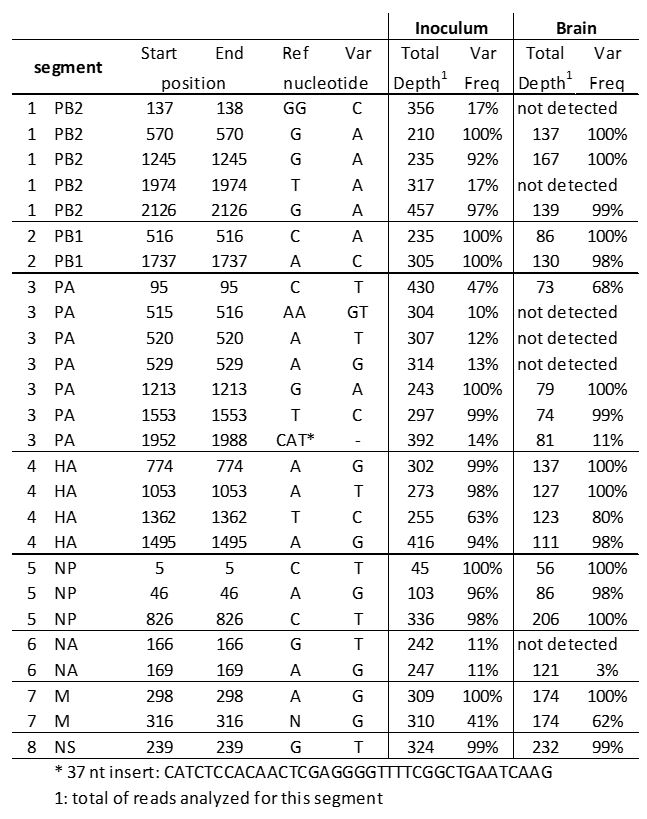

Supplement: S1 Table — (JPG) [file ppat.1009490.s001.jpg]

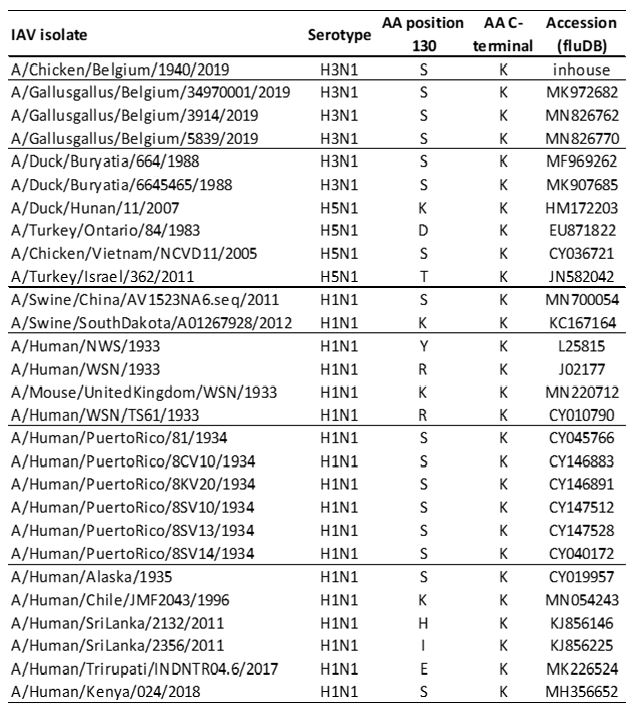

Supplement: S2 Table — (JPG) [file ppat.1009490.s002.jpg]

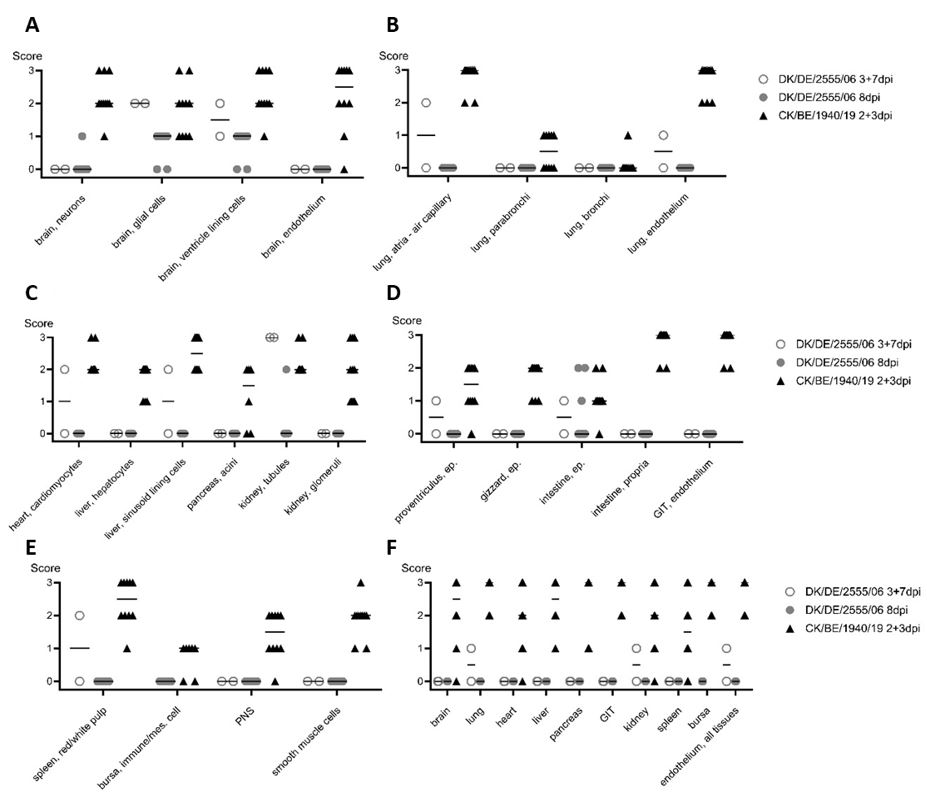

Supplement: S1 Fig — Semi quantitative antigen distribution in the brain (A), lung (B), heart, liver, pancreas and kidney (C), spleen, bursa, peripheral nervous system, smooth muscle cells and endothelium within all tissue (D), gastrointestinal-tract (E), endothelium in selected tissues (F). Dots represent individual animal tissue scores: 0 = no antigen, 1 = focal to oligofocal, 2 = multifocal, 3 = coalescing/diffuse. Bar indicates median, ep. = epithelium, PNS = peripheral nervous system, GIT = gastrointestinal tract. (JPG) [file ppat.1009490.s003.jpg]

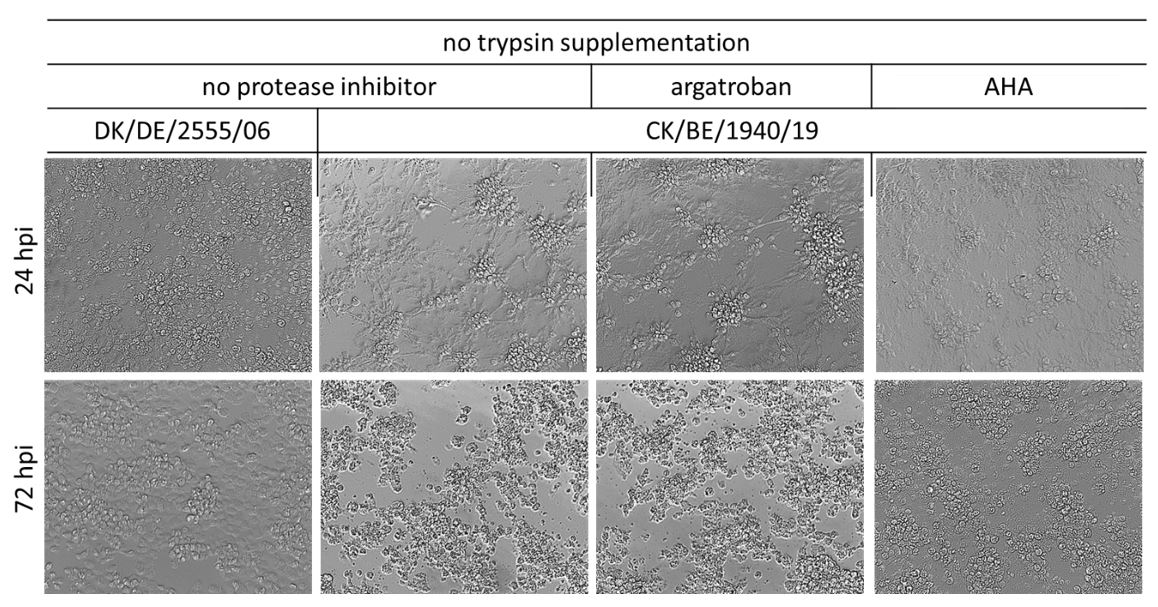

Supplement: S2 Fig — While the LPAIV reference virus DK/DE/2555/06 did not cause visible CPE without trypsin supplementation, the CK/BE/1940/19 H3N1 induced pronounced CPE with complete lysis of the cell layer. CK/BE/1940/19-induced CPE is totally blocked when cultivated in the presence of 6-aminohexanoic acid (AHA), but not with argatroban supplementation. (JPG) [file ppat.1009490.s004.jpg]

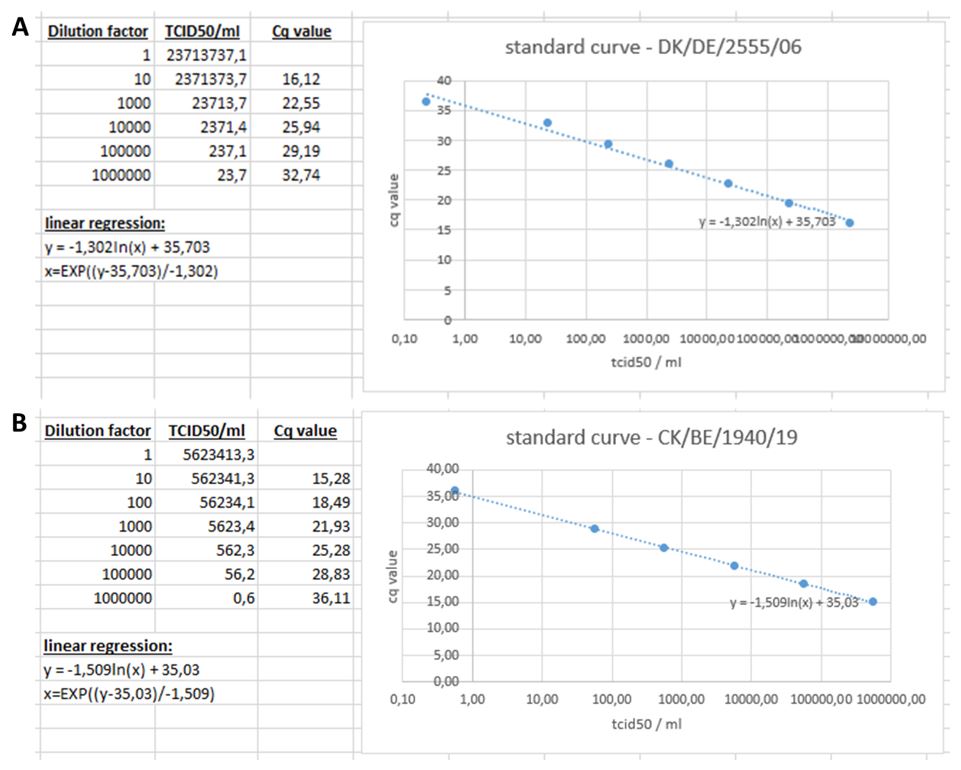

Supplement: S3 Fig — Standard RT-qPCR curve used for infectious virus equivalent (VE) calculation of (A) DK/DE/2555/06 or (B) CK/BE/1940/19 samples. (JPG) [file ppat.1009490.s005.jpg]

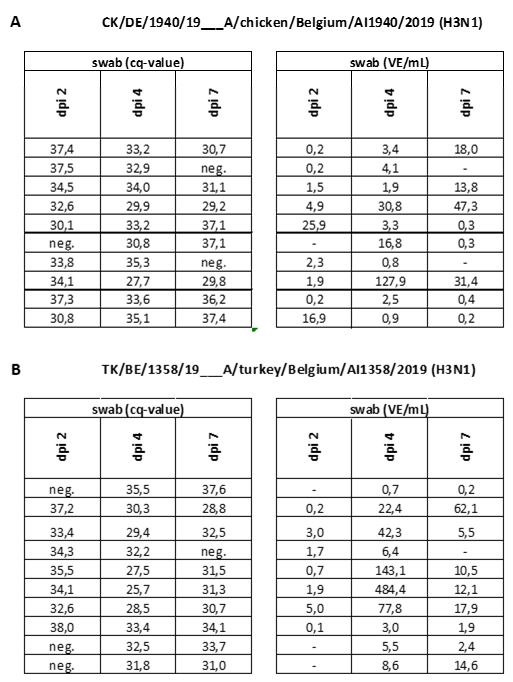

Supplement: S4 Fig — (JPG) [file ppat.1009490.s006.jpg]

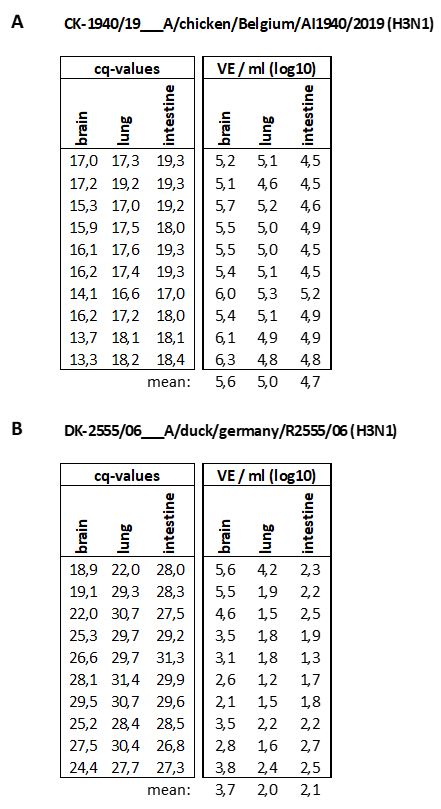

Supplement: S5 Fig — (JPG) [file ppat.1009490.s007.jpg]

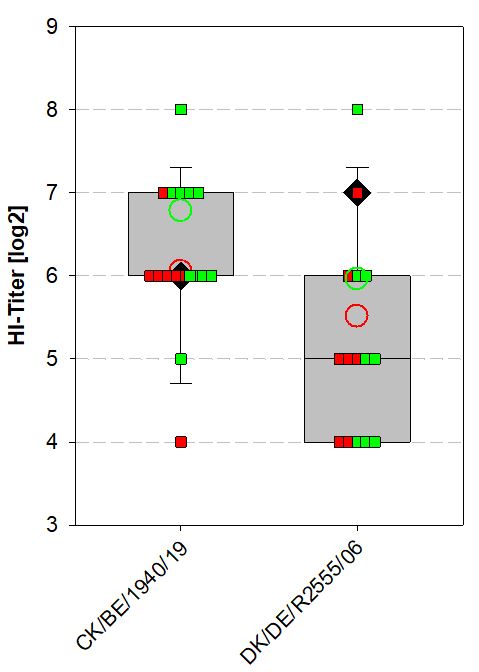

Supplement: S6 Fig — Minor antigenic difference between homologous Belgium strain (CK/BE/1940/19) or heterologous wild bird AIV H3N1 (DK/DE/R2555/06) was observed by HI when sera from chicken inoculated for the IVPI experiment with either CK/BE/1949/19 (n = 9, red) or TK/BE/1358/19 (n = 7, green), obtained 21 weeks after infection. Beside individual values (□) and arithmetic means (○) of the groups, boxblots are representing results of combined results of both groups. No statistically significant difference were evident between infected groups (P = 0,142, t-test, Sigma Plot, Systat Software). A homologues reference serum to DK/DE/R2555/06 (◊) is derived from an immunized chicken and is producing reciprocal results of HI titer (log2) of 7 with homologous DK/DE/R2555/06 antigen vs. HI titer (log2) of 6 with heterologous CK/BE/1940/19. (JPG) [file ppat.1009490.s008.jpg]
